# Quantum Otto heat engine with Pöschl–Teller potential in contact with coherent thermal bath

**DOI:** 10.1038/s41598-023-37681-1

**Published:** 2023-06-29

**Authors:** Sajjad Hashemi Abasabadi, Sayyed Yahya Mirafzali, Hamid Reza Baghshahi

**Affiliations:** Department of Physics, Faculty of Science, Vali-e-Asr University of Rafsanjan, Rafsanjan, Iran

**Keywords:** Optics and photonics, Physics

## Abstract

Work and efficiency of quantum Otto heat engines (QOHEs) can increase by using non-thermal baths or by inhomogeneous scaling of energy levels of the working substance. Given these points, at first, we construct the coherent thermal state for a trigonometric Pöschl–Teller (PT) potential. Then using a particle in this potential, which has unequally spaced energy levels, as a working substance, we investigate the work extraction and the efficiency of QOHEs that operates between cold and hot coherent thermal baths. The results show that changing the PT potential parameters in the adiabatic processes of QOHE, which causes an inhomogeneous shift in energy levels or/and make use of the hot coherent thermal bath, improve work extraction and efficiency of QOHE relative to the classical counterpart.

## Introduction

Classical heat engines are machines which convert thermal energy into work by using the heat flow between hot and cold baths, usually in a thermodynamical cycle. One principle point is that the efficiency of classical heat engines, defined as the ratio of the extracted work to the invested heat, cannot exceed the Carnot bound^1^.

Quantum heat engines, which use quantum matter as their working substance, are defined in similarity to their classical counterparts, with defining the quantum versions of thermodynamical processes that include in their cycle^2^. These engines can produce greater work and efficiency from the thermal energy baths as compared to classical ones^3–6^. Also, they can operate near the classical Carnot limit^7^. Recent researches in the field of quantum heat engines include performing quantum Otto cycles on single trapped ions or atoms^8,9^, optomechanical setups^10,11^, or driven superconducting qubits^12^.

Under some conditions, in quantum heat engines, the work and efficiency can be increased. For example, in the quantum Otto cycle, if the energy levels of working substance are scaled inhomogeneously in the adiabatic processes, then the efficiency of the QOHE can exceed its classical counterparts^5,6^. Given this result, it can be deduced that the efficiency of quantum heat engines, which use quantum systems with unequally spaced energy levels, potentially can be higher than their equivalent classical heat engines, because altering the parameters of these systems can create an inhomogeneous shift of energy levels. As an example, PT potential which is used as confining potential of diatomic molecules^13,14^ and quantum dots^15–18^, unlike the harmonic oscillator have unequally spaced energy levels. Therefore, using this potential as a working substance, can improve the efficiency of quantum heat engines.

Another attractive condition that can improve the performance of the quantum heat engine is that non-thermal baths to be used in the heat engines cycle^19^. Specifically, it has been shown that by using a harmonic oscillator as a working substance, in QOHE that operates between cold and hot coherent thermal (squeezed thermal) baths, the efficiency can even reach one^20,21^.

On the other hand, the concept of coherent states of the harmonic oscillator has been generalized to arbitrary potentials with different methods^22^. These methods have also been applied to PT potential and the coherent states for this potential have been defined^22–25^. Using these results, we can construct thermal coherent states of PT potential.

According to the previous statements, in this paper at the beginning, we construct the thermal coherent state for a trigonometric PT potential. Then using a particle in this potential as a working substance, we investigate the work extraction and the efficiency of the QOHE that operates between cold thermal and hot coherent thermal baths.

The reminder of this paper is planned as follows. In “Thermal coherent state of PT potential” the thermal coherent state of the PT potential is constructed. In “Classical Otto heat engine” and “Quantum Otto heat engine”, the classical Otto heat engine (COHE) and QOHE are investigated, respectively. In detail, “Quantum Otto heat engine” includes two parts, in “Quantum Otto heat engine with two thermal baths” (“Quantum Otto heat engine with coherent thermal bath”) of this section a QOHE that operates between a cold thermal bath and a hot thermal (thermal coherent) bath is examined. Finally, “Conclusion” is closed by summary and conclusion remarks.

## Thermal coherent state of PT potential

The well-known coherent states of quantum harmonic oscillators have some attractive properties that are considered as the definition of these states. Based on each one of these properties, the definition of coherent states has been generalized to a system other than the harmonic oscillator^22–26^.

In this paper, we use the definition of coherent states as displaced ground (vacuum) states, i.e. the coherent states $|\alpha \rangle$ can be created from the ground state $|0\rangle$ by applying the displacement operator $D(\alpha )=\mathrm{exp}(\alpha {a}^{\dagger}-{\alpha }^{*}a)$, $|\alpha \rangle =D(\alpha )|0\rangle$, where $a$ and ${a}^{\dagger}$ are annihilation and creation operators of the harmonic oscillator, respectively. Also, based on this definition, thermal coherent states for the harmonic oscillator $\rho (\alpha ,\beta )$ are defined as displaced thermal states, that are:

1 $$\rho (\alpha ,\beta )=\frac{1}{Z}D(\alpha )\mathrm{exp}(-\beta H){D}^{\dagger}(\alpha )=\frac{1}{Z}\sum_{n=0}^{\infty }\mathrm{exp}(-\beta {E}_{n})D(\alpha )|n\rangle \langle n|{D}^{\dagger}(\alpha ),$$

where $\beta =\frac{1}{{k}_{B}T}$, $H$ is the Hamiltonian of the system, $Z={\sum }_{n=0}^{\infty }\mathrm{exp}(-\beta {E}_{n})$ is the partition function and ${E}_{n}$ are eigenvalues of $H$ (in the above equation usually, $D(\alpha )|n\rangle$ is called displaced number states). So, based on the above definition, in order to define coherent and thermal coherent states for an arbitrary potential, it is necessary that at first, one construct the ladder operators for the Hamiltonian of the system and then using them, create the generalized displacement operator for the system. By application of this generalized displacement operator upon the ground state or thermal state of the system, the coherent and thermal coherent states can be obtained.

Now, following this procedure, we construct the thermal coherent states for the trigonometric PT potential, $V(x)={U}_{0}{\mathrm{tan}}^{2}(ax)$ with eigenvalues ${E}_{n,\lambda }=\frac{{{\hslash }}^{2}{a}^{2}}{2m}({n}^{2}+2n\lambda +\lambda )$, where $m$ is the mass of the particle, ${U}_{0}$ is the parameter which determines the potential’s strength, $a=\pi /2L$ where $2L$ is the range of the potential, and $\lambda$ is the dimensionless parameter which determines according to the relation $\lambda (\lambda -1)=\frac{2m{U}_{0}}{{{\hslash }}^{2}{a}^{2}}$^23^. The ladder operators $b$, ${b}^{\dagger}$ and generalized displacement operator $D(\alpha )=\mathrm{exp}(\alpha {b}^{\dagger}-{\alpha }^{*}b)$ for this potential, were constructed in^22^. By writing $\alpha =|\alpha |{e}^{i\Phi }$ and defining $\zeta ={e}^{i\Phi }\mathrm{tanh}(|\alpha |/\sqrt{2\lambda })$, the generalized displacement operator for PT potential can be written as:

2 $${D}_{D}(\zeta )={e}^{(\zeta \sqrt{2\lambda }{b}^{\dagger})}(1-|\zeta {|}^{2}{)}^{\lambda {b}_{0}}{e}^{(-{\zeta }^{*}\sqrt{2\lambda }b)},$$

where ${b}_{0}=1+\frac{n}{\lambda }$. Using the following relations:

3 $$b|n,\lambda \rangle =\sqrt{\frac{n(2\lambda +n-1)}{2\lambda }}|n-1,\lambda \rangle , {b}^{\dagger}|n,\lambda \rangle =\sqrt{\frac{(n+1)(2\lambda +n)}{2\lambda }}|n+1,\lambda \rangle ,$$

where $|n,\lambda \rangle$ are the eigenvectors of the PT potential, and applying ${D}_{D}(\zeta )$ to the vacuum state $|0,\lambda \rangle$, the coherent state for the PT potential $|\zeta \rangle$ has been obtained as follows:

4 $$|\zeta \rangle ={D}_{D}(\zeta )|0,\lambda \rangle =(1-|\zeta {|}^{2}{)}^{\lambda }\sum_{n=0}^{\infty }\sqrt{\frac{\Gamma (n+2\lambda )}{n!\Gamma (2\lambda )}}{\zeta }^{n}|n,\lambda \rangle .$$

Now, we construct the displaced number states $|\zeta ,n\rangle$. After some calculations, we obtain:

5 $$|\zeta ,n\rangle ={D}_{D}(\zeta )|n,\lambda \rangle =\sum_{p=0}^{n}\sum_{k=0}^{p}{F}_{n,p,k}(\zeta )|p\rangle +\sum_{p>n}^{\infty }\sum_{k=p-n}^{p}{F}_{n,p,k}(\zeta )|p\rangle ,$$

6 $${F}_{n,p,k}(\zeta )=(1-|\zeta {|}^{2}{)}^{\lambda }(1-|\zeta {|}^{2}{)}^{p-k}\times \frac{(-{\zeta }^{*}{)}^{n-p+k}(\zeta {)}^{k}}{(n-p+k)!k!}\frac{(n!p!\Gamma (2\lambda +n)\Gamma (2\lambda +p){)}^\frac{1}{2}}{(p-k)!\Gamma (2\lambda +p-k)}.$$

Having $|\zeta ,n\rangle$ and eigenvalues ${E}_{n,\lambda }$ of the PT potential, we can define the thermal coherent states for this potential as the following form:

7 $$\rho (\zeta ,\lambda ,\beta )=\frac{1}{Z}\sum_{n=0}^{\infty }\mathrm{exp}(-\beta {E}_{n,\lambda })|\zeta ,n\rangle \langle \zeta ,n|,$$

where $Z={\sum }_{n=0}^{\infty }\mathrm{exp}(-\beta {E}_{n,\lambda })$ is the partition function. In the “Quantum Otto heat engine with coherent thermal bath”, we will use $\rho (\zeta ,\lambda ,\beta )$ for calculation of the work and efficiency in the QOHE.

## Classical Otto heat engine

In the classical thermodynamics, the Otto cycle is provided as a generic model for spark-ignition internal combustion engines^1^.

**Figure 1:**
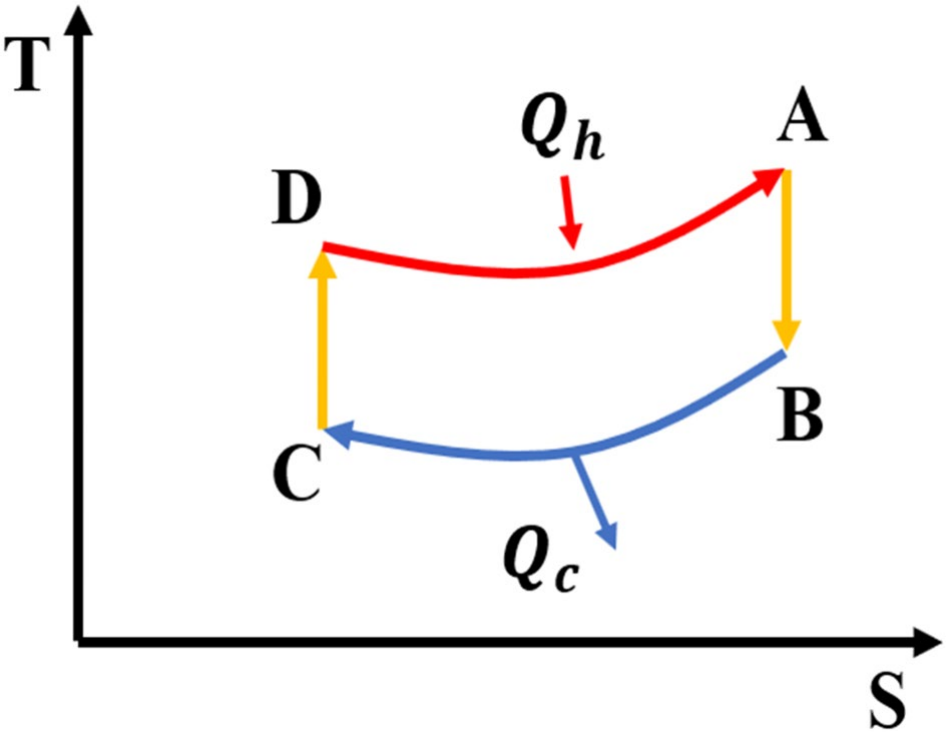
The Temperature-Entropy diagram of the classical Otto cycle. ${Q}_{h}$ is the heat absorbed from a hot thermal bath and ${Q}_{c}$ is the heat released in cold thermal bath.

The operation of the Otto cycle on a T–S diagram is shown in Fig. 1.

This cycle is composed of four strokes, including:

- Stroke $C\to D$: adiabatic compression process in which the working substance decoupled from the cold bath. During this stroke by altering parameter $a$ in the PT potential the volume of system changes, such that the Hamiltonian is changed adiabatically from ${H}_{c}$ to ${H}_{h}$.
- Stroke $D\to A$: isochoric heat absorption process in which, in contact with hot thermal bath, heat is absorbed (${Q}_{h}$) at constant volume.
- Stroke $A\to B$: adiabatic expansion process in which the working substance decoupled from the hot bath. During this stroke by altering parameter $a$ in the PT potential the volume of system changes, such that the Hamiltonian is changed adiabatically from ${H}_{h}$ to ${H}_{c}$.
- Stroke $B\to C$: isochoric heat rejection process in which, in contact with cold thermal bath, heat is released (${Q}_{c}$) at constant volume.

The efficiency of the Otto heat engine is defined as follow:

8 $${\eta }_{Otto}=\frac{{W}_{out}}{{Q}_{h}}.$$

In this relation, ${W}_{out}=({U}_{B}-{U}_{A})+({U}_{D}-{U}_{C})$ is the total work output of the COHE and ${Q}_{h}=({U}_{A}-{U}_{D})$ is the heat absorbed from a hot thermal bath in the stroke $D\to A$ where ${U}_{i}(i=A,B,C,D)$ is the internal energy of the working substance in the point $i$.The explicit expression for $U$ and the efficiency of the COHE for PT potential is represented in the Appendix A in Supplementary Information.

The strength and range of the PT potential can be changed by altering parameters ${U}_{0}$, and $a$, respectively. In the COHE the efficiency and work can be obtained when, during the adiabatic processes, the volume of a system changes in the stroke $A\to B$ and the stroke $C\to D$. If ${U}_{0}$ is changed, the volume does not changes, so the efficiency of the COHE is independent of ${U}_{0}$. Nevertheless, efficiency is dependent on $a$ because changing $a$ causes the volume of the system changes.

**Figure 2:**
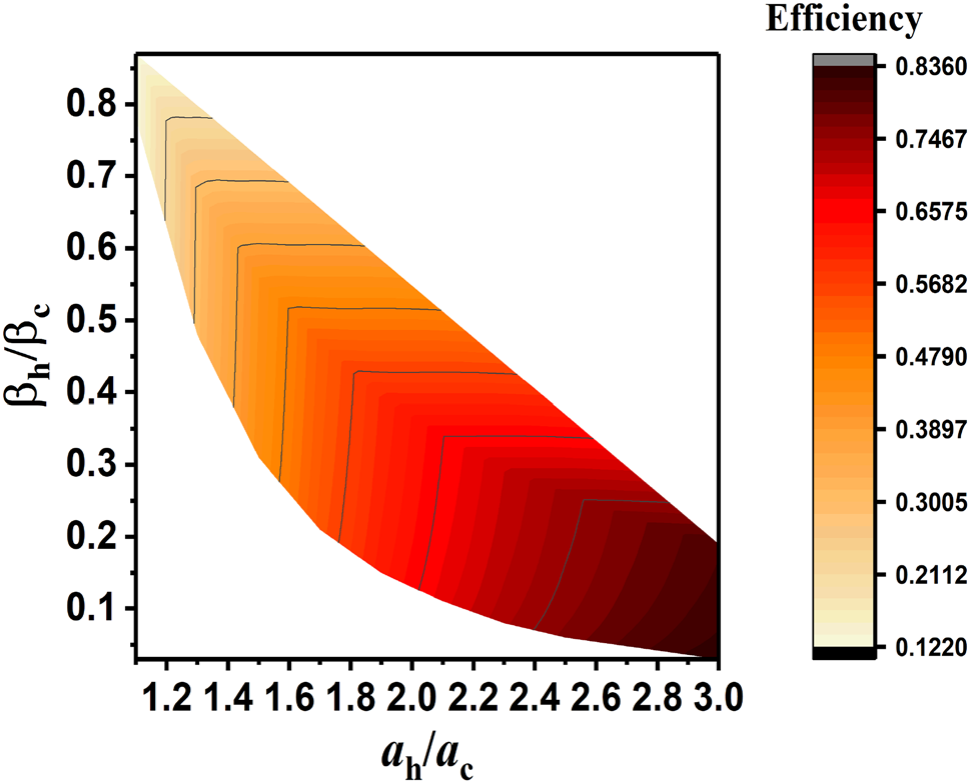
The efficiency of the COHE in contact with thermal bath. ${a}_{c}$ and ${a}_{h}$ are the values of $a$ when the system is in contact with cold and hot thermal baths, and ${T}_{c}$ and ${T}_{h}$ are the temperature of cold and hot thermal baths, respectively.

In Fig. 2, the efficiency of the COHE cycle with ${U}_{0}=1$, in terms of ${\beta }_{h}/{\beta }_{c}={T}_{c}/{T}_{h}$ (when ${T}_{c}$ and ${T}_{h}$ are the temperature of cold and hot thermal baths, respectively), and ${a}_{h}/{a}_{c}$ (where ${a}_{c}$ and ${a}_{h}$ are the value of $a$ when the system is in contact with cold and hot thermal baths, respectively) is represented. In this plot, it can be seen that by raising ${a}_{h}/{a}_{c}$ and also by lowering ${\beta }_{h}/{\beta }_{c}$ (that means the difference between temperature of the hot and cold thermal baths increases) efficiency of the COHE increases. On the other hand, the efficiency of the COHE does not exceed the Carnot bound which is equal to ${\eta }_{C}=1-\frac{{\beta }_{h}}{{\beta }_{c}}$.

## Quantum Otto heat engine

In this section, we use a particle in the PT potential as the working substance of a QOHE. We consider two different cases separately. In the “Quantum Otto heat engine with two thermal baths” (“Quantum Otto heat engine with coherent thermal bath”), we consider a QOHE operating between a cold thermal bath and a hot thermal bath (hot coherent thermal bath). In each case, we investigate the work extraction and the efficiency of the engine.

### Quantum Otto heat engine with two thermal baths

**Figure 3:**
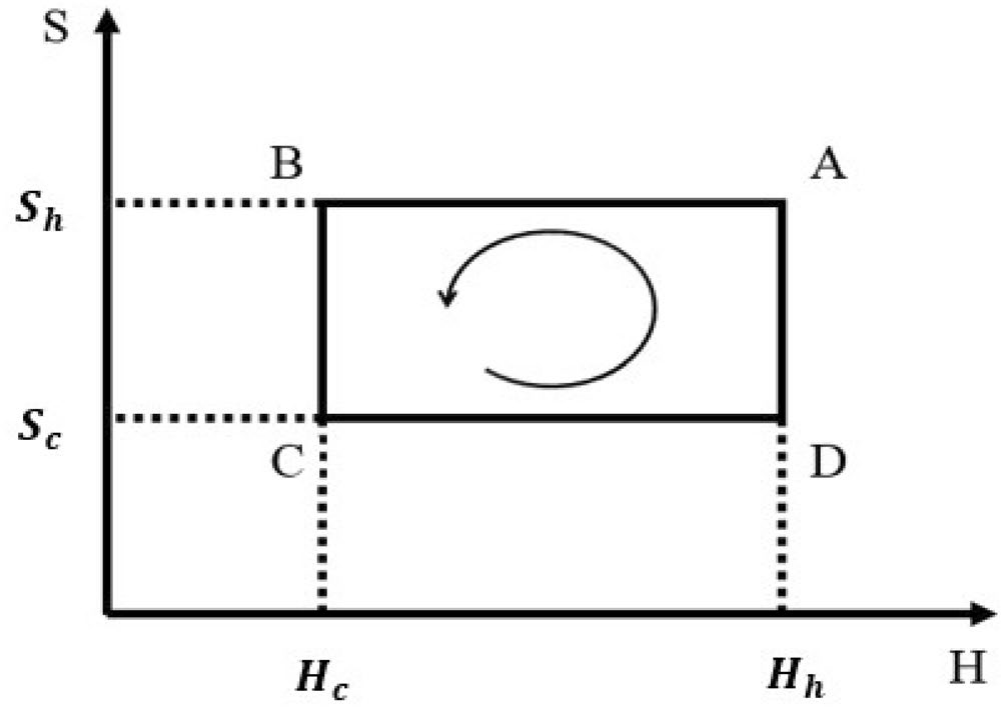
The Entropy-Hamiltonian (S–H) diagram of the quantum Otto cycle, which consists of two quantum isochoric processes ($D\to A,B\to C$) and two quantum adiabatic processes ($A\to B,C\to D$). During the quantum isochoric processes, the energy levels remain constant, but, the occupation probability of energy levels changes, while in the quantum adiabatic processes, the occupation probability of energy levels is kept constant and merely the energy levels scale adiabatically.

QOHE operating between a cold thermal bath at inverse temperature ${\beta }_{c}$ and a hot thermal bath at inverse temperature ${\beta }_{h}$ is a four strokes cycle (Fig. 3) consisting of two isochoric processes (strokes $D\to A$ and $B\to C$) and two adiabatic processes (strokes $C\to D$ and $A\to B$). In isochoric processes, the potential is kept constant while the working substance equilibrates with the cold or the hot bath. During these processes, no work is done and the system only exchanges heat. In adiabatic processes, the working substance is decoupled from thermal baths and it does not exchange heat with the environment. So, from the first law of thermodynamics, we deduce that the total work extracted in the cycle is given by ${W}_{out}={Q}_{c}+{Q}_{h}$ where ${Q}_{c}=\mathrm{Tr}(({\rho }_{C}-{\rho }_{B}){H}_{c})$ and ${Q}_{h}=\mathrm{Tr}(({\rho }_{A}-{\rho }_{D}){H}_{h})$ are heat exchanged by the cold and hot thermal baths, respectively, such that ${H}_{c}$ and ${H}_{h}$ are the Hamiltonian of the system in contact with cold and hot thermal baths. Also, ${\rho }_{A}$, ${\rho }_{B}$, ${\rho }_{C}$ and ${\rho }_{D}$ are the density matrix of the working substance in the points $A$, $B$, $C$, and $D$, respectively.

**Figure 4:**
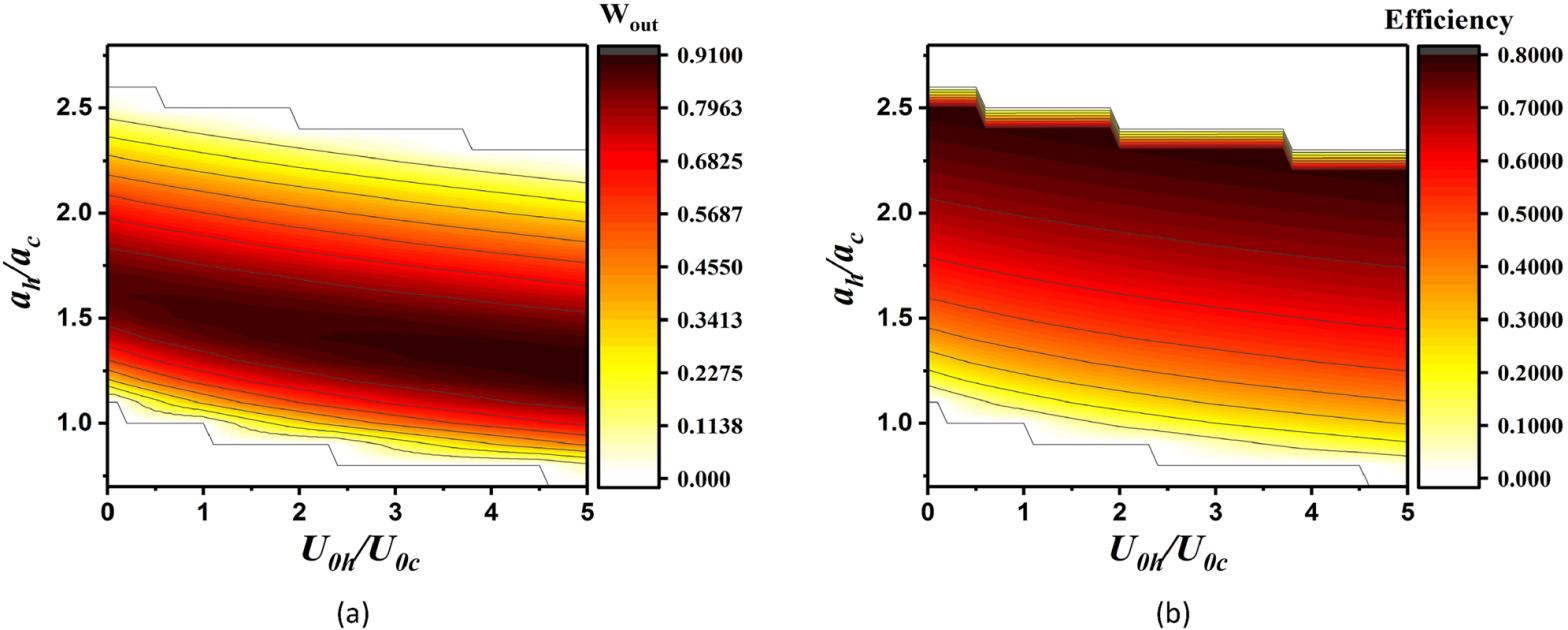
(**a**) The total work output (in units of ${\hslash }\omega ={{\hslash }}^{2}\lambda {a}^{2}/m$) of the QOHE in contact with thermal bath for parameter ${\beta }_{h}=0.2{\beta }_{c}$ in terms of ${U}_{0h}/{U}_{0c}$ and ${a}_{h}/{a}_{c}$. ${U}_{0i}$, $i=h,c$, is potential’s strength in contact with hot, $i=h$, or cold, $i=c$, thermal bath. (**b**) The efficiency of the QOHE in contact with thermal bath for parameter ${\beta }_{h}=0.2{\beta }_{c}$.

In Fig. 4, the total work output and efficiency of the QOHE, ${\eta }_{Otto}=\frac{{W}_{out}}{{Q}_{h}}$, in contact with cold and hot thermal baths are plotted. From these figures it can be seen that if ${U}_{0}$ (where ${U}_{0i}$, $i=h,c$, is potential’s strength in contact with hot, $i=h$, or cold, $i=c$, thermal bath) is changed, in the QOHE, the work and efficiency will be non zero. Depending on the ${a}_{h}/{a}_{c}$ and ${U}_{0h}/{U}_{0c}$, quantum Otto cycle presents different regimes of operation. It operates as the regular heat engine where ${Q}_{c}\le 0$, ${Q}_{h}\ge 0$ and ${W}_{out}={Q}_{c}+{Q}_{h}\ge 0$, however it can operate as a refrigerator where ${Q}_{c}\ge 0$, ${Q}_{h}\le 0$ and ${W}_{out}={Q}_{c}+{Q}_{h}\le 0$ (approximately for ${a}_{h}/{a}_{c}\gtrsim 2.7$), or as heater^5^ where ${Q}_{c}\le 0$, ${Q}_{h}\ge 0$ and ${W}_{out}={Q}_{c}+{Q}_{h}\le 0$ (approximately for ${a}_{h}/{a}_{c}\lesssim 0.5$). In this paper, we restrict our study to the regular heat engine operation regimes, so in our calculations anywhere we obtained ${W}_{out}\le 0$, we put ${W}_{out}=0$.

**Figure 5:**
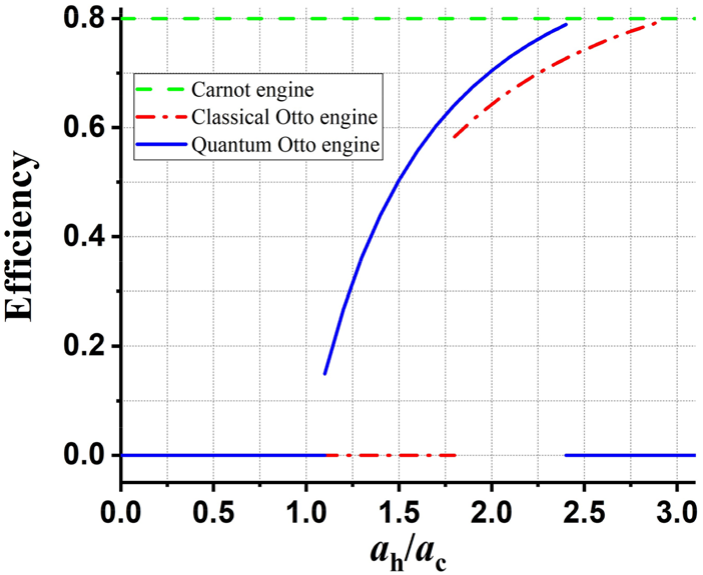
The efficiency of the Carnot, COHE, and QOHE for parameters ${\beta }_{h}=0.2{\beta }_{c}$, and ${U}_{0}={U}_{0c}={U}_{0h}=1$.

In Fig. 5, the efficiency of the Carnot, COHE, and QOHE that are in contact with the thermal bath is displayed in terms of ${a}_{h}/{a}_{c}$ for parameters ${U}_{0}=1$ and ${\beta }_{h}=0.2{\beta }_{c}$. According to this plot, it can be seen that in interval $1.8\le {a}_{h}/{a}_{c}\le 2.4$, the efficiency of the QOHE is more than the efficiency of its classical counterpart. Also, it can be seen that in interval $1.1\le {a}_{h}/{a}_{c}<1.8$, the efficiency of QOHE is not zero, while in this interval which means low-volume changes, the COHE is zero. On the other hand, in interval $2.4<{a}_{h}/{a}_{c}\le 2.9$, the efficiency of QOHE is zero, while in this interval, the COHE have efficiency although for ${a}_{h}/{a}_{c}>2.9$ the efficiency of COHE and QOHE are zero. Also, it is notable that in $1.1<{a}_{h}/{a}_{c}$ and ${a}_{h}/{a}_{c}>1.8$ for the QOHE and also, in $1.8<{a}_{h}/{a}_{c}$ and ${a}_{h}/{a}_{c}>2.4$ for the COHE, we set the efficiency to be zero because this efficiency is not inside the area of the heat engine. Finally, it is worth noting that the efficiency of Otto heat engines, classical or quantum, can not exceed the Carnot bound.

### Quantum Otto heat engine with coherent thermal bath

In this subsection, we consider a QOHE operating between two baths: a cold thermal bath at inverse temperature ${\beta }_{c}$ and a hot coherent thermal bath at ${\beta }_{h}\le {\beta }_{c}$ with coherent parameter $\alpha$.

The Otto cycle is composed of four strokes that connect different states of the system in Fig. 3A–D. We start with our system in point C. In this point working substance put in equilibrium with cold thermal bath. The Hamiltonian is ${H}_{c}=\frac{{p}^{2}}{2m}+{V}_{c}(x)$ where ${V}_{c}(x)={U}_{0c}{\mathrm{tan}}^{2}({a}_{c}x)$ and density matrix is ${\rho }_{C}={Z}_{c}^{-1}\mathrm{exp}(-{\beta }_{c}{H}_{c})$ where ${Z}_{c}=\mathrm{Tr}(\mathrm{exp}(-{\beta }_{c}{H}_{c}))$ is partition function. The first stroke $C\to D$ is an adiabatic process in which the working substance decoupled from the bath. During this stroke the Hamiltonian is changed adiabatically from ${H}_{c}$ to ${H}_{h}=\frac{{p}^{2}}{2m}+{V}_{h}(x)$ where ${V}_{h}(x)={U}_{0h}{\mathrm{tan}}^{2}({a}_{h}x)$ such that occupation probability of system’s energy levels does not change. The state of the working substance at point D can be written ${\rho }_{D}={Z}_{c}^{-1}{\sum }_{n}\mathrm{exp}(-{\beta }_{c}{E}_{n,\lambda ,c})|n,\lambda {\rangle }_{hh}\langle n,\lambda |$ where $|n,\lambda {\rangle }_{h}$ are eigenstates of ${H}_{h}$. In this stroke there is not heat exchange with the bath and also entropy is constant, while the work extracted during this stroke is ${W}_{C\to D}=\mathrm{Tr}({\rho }_{D}{H}_{h})-\mathrm{Tr}({\rho }_{C}{H}_{c})$. The second stroke $D\to A$ is an isochoric process in which the Hamiltonian of the working substance is kept fixed at ${H}_{h}$. In this stroke working substance put in contact with a hot coherent thermal bath, until its state change to thermal coherent state ${\rho }_{A}=D(\alpha ){Z}_{h}^{-1}\mathrm{exp}(-{\beta }_{h}{H}_{h}){D}^{\dagger}(\alpha )$ where ${Z}_{h}=\mathrm{Tr}(\mathrm{exp}(-{\beta }_{h}{H}_{h}))$ is partition function. The explicit explanation for this process is represented in the Appendix B in Supplementary Information. In this stroke, no work is done and the energy changes are equal to ${Q}_{D\to A}=\mathrm{Tr}(({\rho }_{A}-{\rho }_{D}){H}_{h})$. In third stroke, at first the state of the system by unitary transformation converted to the thermal state ${\rho }_{th}={D}^{\dagger}(\alpha ){\rho }_{A}D(\alpha )$, and then by an adiabatic process its Hamiltonian return to its original value ${H}_{c}$. The density matrix in point $B$ is ${\rho }_{B}={Z}_{h}^{-1}{\sum }_{n}\mathrm{exp}(-{\beta }_{h}{E}_{n,\lambda ,h})|n,\lambda {\rangle }_{cc}\langle n,\lambda |$ and also the work extraction in this stroke is ${W}_{A\to B}=\mathrm{Tr}({\rho }_{B}{H}_{c})-\mathrm{Tr}({\rho }_{A}{H}_{h})$. Finally, the cycle is closed by means of the fourth stroke $B\to C$, which is the second isochoric process in the cycle and the system return to ${\rho }_{C}$ in contact with cold thermal bath. The heat exchange in this stroke is given by ${Q}_{B\to C}=\mathrm{Tr}(({\rho }_{C}-{\rho }_{B}){H}_{c})$.

Now, by use of the above expressions for work and heat, the efficiency of the QOHE can be calculated as follows:

9 $$\eta =\frac{{Q}_{D\to A}+{Q}_{B\to C}}{{Q}_{D\to A}}.$$

It is worth noting that to calculate the efficiency and work, it is necessary to obtain the average value of energy for the four points of the Otto cycle. As previously mentioned in point A, the working substance is in contact with the hot coherent thermal bath. At this point the density matrix is ${\rho }_{A}=D(\alpha ){Z}_{h}^{-1}\mathrm{exp}(-{\beta }_{h}{H}_{h}){D}^{\dagger}(\alpha )$. The average value of energy in this point can be calculated as follows:

10 $$\begin{array}{ll}\langle {H}_{h}{\rangle }_{A}=\mathrm{Tr}({\rho }_{A}{H}_{h})& =\sum_{n=0}^{\infty }\frac{{e}^{-\frac{{E}_{n,h}}{{k}_{B}{T}_{h}}}}{{Z}_{h}}(\sum_{m=0}^{n}{E}_{n,h}|\sum_{k=0}^{m}{F}_{n,m,k}(\zeta ){|}^{2}+\sum_{m>n}^{\infty }{E}_{m,h}|{F}_{n,m,k}(\zeta ){|}^{2})\\ & =(\frac{1}{2}+\frac{1}{2}{\mathrm{cosh}}^{2}(\sqrt{\frac{2}{\lambda }}|\alpha |)+{\mathrm{sinh}}^{2}(\sqrt{\frac{2}{\lambda }}|\alpha |))\mathrm{Tr}({Z}_{h}^{-1}\mathrm{exp}(-{\beta }_{h}{H}_{h}){H}_{h})\\ & +\frac{{\hslash }^{2}{a}^{2}}{2m}({\lambda }^{2}-\lambda ){\mathrm{sinh}}^{2}(\sqrt{\frac{2}{\lambda }}|\alpha |).\end{array}$$

The derivation of the Eq. (10) is explained in the Appendix C in Supplementary Information, in details. According to this equation, the heat absorbed by cycle in the isochoric process ($D\to A$) is as follows:

11 $$\begin{array}{ll}{Q}_{D\to A}=& (\frac{1}{2}+\frac{1}{2}{\mathrm{cosh}}^{2}(\sqrt{\frac{2}{\lambda }}|\alpha |)+{\mathrm{sinh}}^{2}(\sqrt{\frac{2}{\lambda }}|\alpha |))\mathrm{Tr}({Z}_{h}^{-1}\mathrm{exp}(-{\beta }_{h}{H}_{h}){H}_{h})\\ & +\frac{{\hslash }^{2}{a}^{2}}{2m}({\lambda }^{2}-\lambda ){\mathrm{sinh}}^{2}(\sqrt{\frac{2}{\lambda }}|\alpha |)-Tr({\rho }_{D}{H}_{h}),\end{array}$$

now by substitution the Eq. (11) in the Eq. (9), the efficiency of the QOHE with coherent thermal bath can be written as follows:

12 $$\eta =1+\frac{{Q}_{B\to C}}{(\frac{1}{2}+\frac{1}{2}{\mathrm{cosh}}^{2}(\sqrt{\frac{2}{\lambda }}|\alpha |)+{\mathrm{sinh}}^{2}(\sqrt{\frac{2}{\lambda }}|\alpha |))\mathrm{Tr}({Z}_{h}^{-1}\mathrm{exp}(-{\beta }_{h}{H}_{h}){H}_{h})+\frac{{\hslash }^{2}{a}^{2}}{2m}({\lambda }^{2}-\lambda ){\mathrm{sinh}}^{2}(\sqrt{\frac{2}{\lambda }}|\alpha |)-\mathrm{Tr}({\rho }_{D}{H}_{h})},$$

which can be compared with efficiency of the QOHE with two thermal baths which is,

13 $$\eta =1+\frac{{Q}_{B\to C}}{\mathrm{Tr}({Z}_{h}^{-1}\mathrm{exp}(-{\beta }_{h}{H}_{h}){H}_{h})-\mathrm{Tr}({\rho }_{D}{H}_{h})}.$$

According to the above description, it can be seen that in the limit $\alpha \to 0$, the efficiency Eq. (12) is identical to Eq. (13). In addition $(\frac{1}{2}+\frac{1}{2}{\mathrm{cosh}}^{2}(\sqrt{\frac{2}{\lambda }}|\alpha |)+{\mathrm{sinh}}^{2}(\sqrt{\frac{2}{\lambda }}|\alpha |))$ is greater than one and $\lambda =(1+\sqrt{1+2{U}_{0}/{a}^{2}})>1$, so the denominator in Eq. (12) is always greater than the denominator in Eq. (13), then the efficiency in Eq. (12) is always greater than the efficiency in Eq. (13).

**Figure 6:**
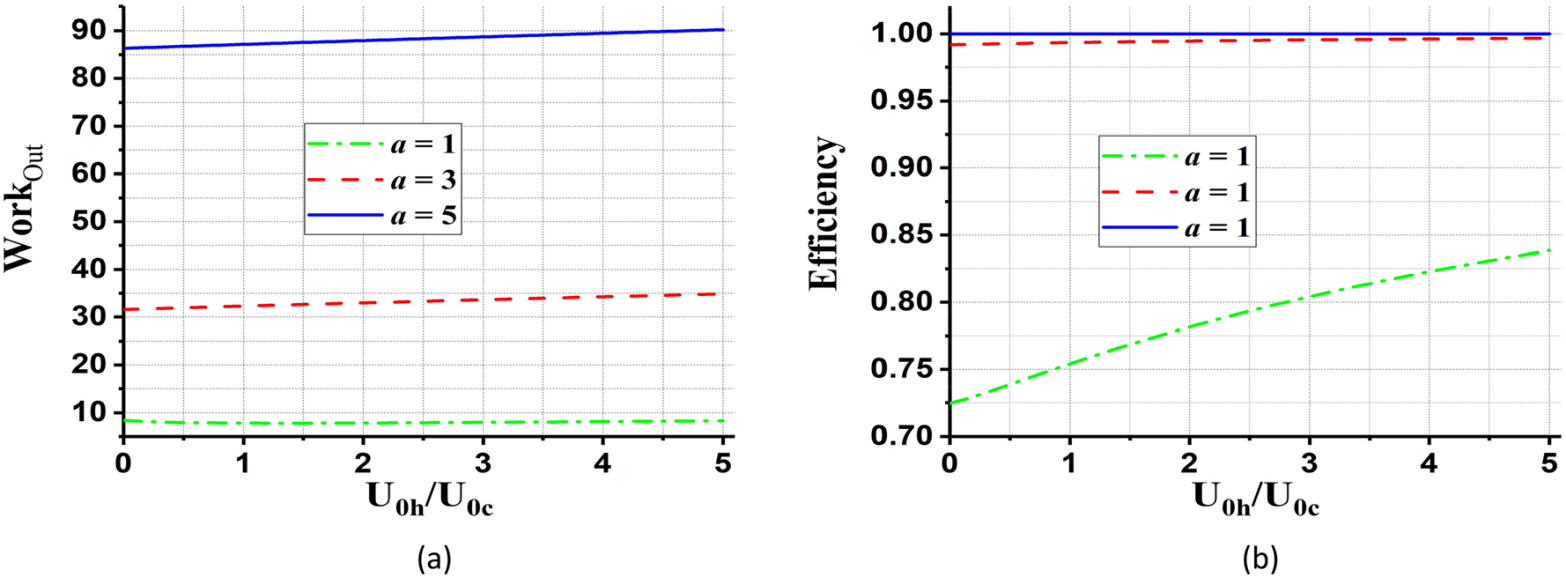
(**a**) The total work output and (**b**) the efficiency (in units of $\hslash \omega ={\hslash }^{2}\lambda {a}^{2}/m$) of the QOHE as a function of ${U}_{0h}/{U}_{0c}$, for different values of $a={a}_{h}={a}_{c}={1,3},5$. We set coherent parameter, $\alpha =1$, and ${\beta }_{h}=0.2{\beta }_{c}$.

In Fig. 6, we plot the total work output and efficiency of the cycle as a function of ${U}_{0h}/{U}_{0c}$ for different values of parameter $a$. As can be seen in these two figures, by increasing $a$, the work and efficiency enhance and even the efficiency reach one.

**Figure 7:**
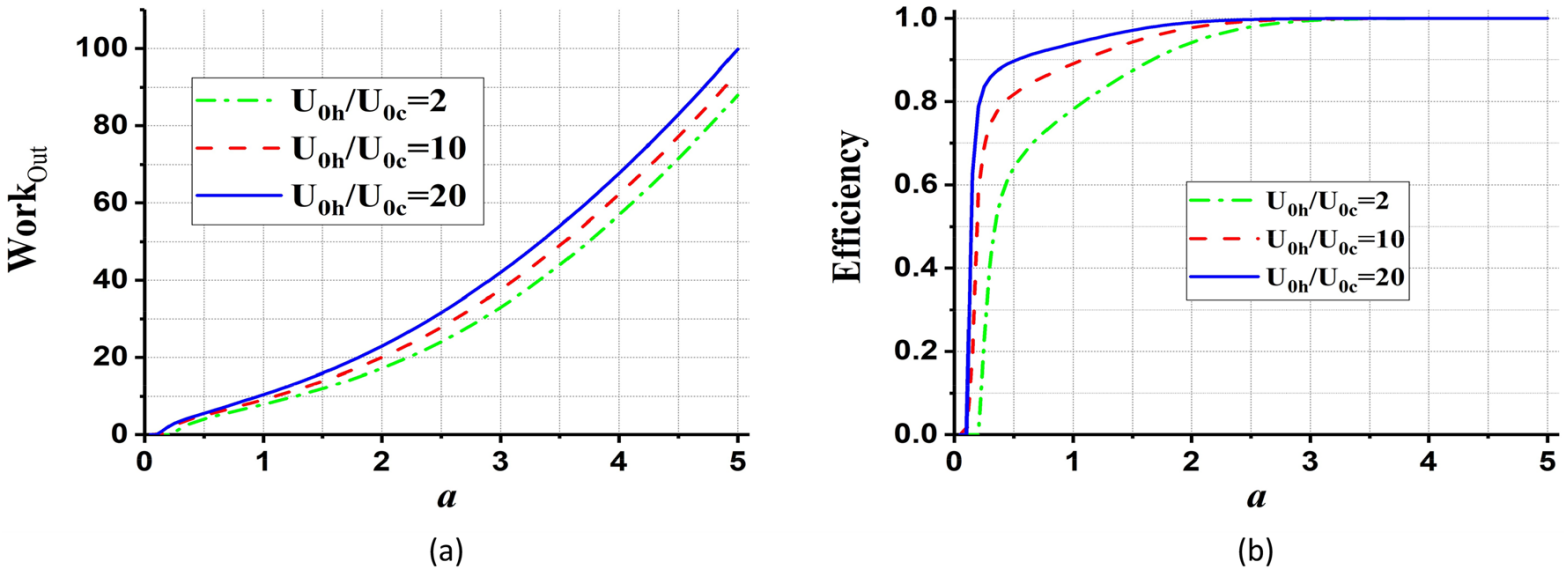
(**a**) The total work output and (**b**) the efficiency of the QOHE as a function of $a={a}_{h}={a}_{c}$ for different values of ${U}_{0h}/{U}_{0c}= {2,10,20}$. We set coherent parameter, $\alpha =1$, and ${\beta }_{h}=0.2{\beta }_{c}$.

In Fig. 7, we have discussed the behavior of total work and efficiency in terms of $a$, for different values of potential’s strength. It is found from Fig. 7 that the total work output from the QOHE is slowly enhanced by increasing the parameter ${U}_{0h}/{U}_{0c}$. Also, it can be seen from Fig. 7 which the efficiency of the QOHE reaches one for different values of ${U}_{0h}/{U}_{0c}$.

**Figure 8:**
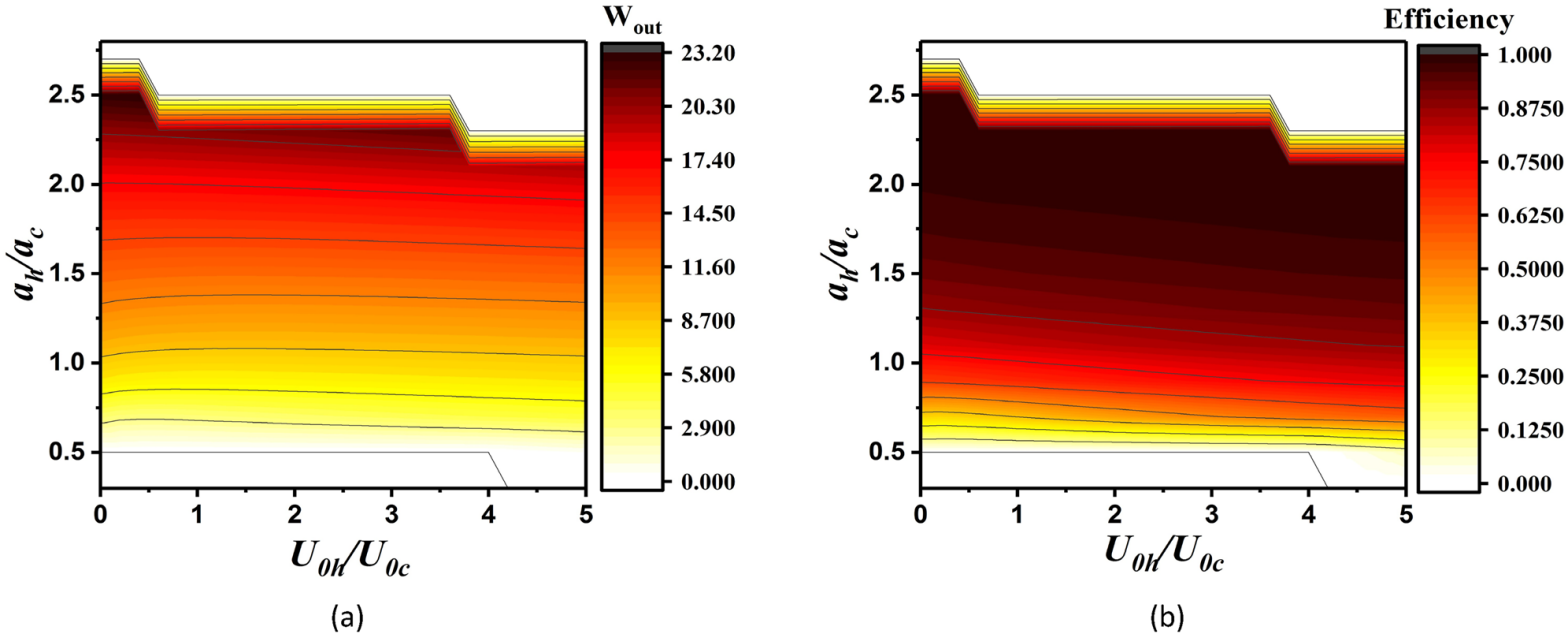
(**a**) The total work output and (**b**) the efficiency (in units of $\hslash \omega ={\hslash }^{2}\lambda {a}^{2}/m$) of the QOHE in contact with the coherent thermal bath for parameters ${\beta }_{h}=0.2{\beta }_{c}$ and $\alpha =1$.

In Fig. 8, the total work output and efficiency of the Otto cycle in terms of ${U}_{0h}/{U}_{0c}$ and ${a}_{h}/{a}_{c}$ with constant value $\alpha =1$ have been represented. In these two figures, it can be observed that for $0.5\lesssim {a}_{h}/{a}_{c}\lesssim 2.7$ with increasing ${a}_{h}/{a}_{c}$ the total work output and efficiency increases. Approximately for ${a}_{h}/{a}_{c}\gtrsim 2.7$, ${Q}_{c}\ge 0$ and ${Q}_{h}\ge 0$ which means that the cycle takes heat from both the hot and cold baths and produces work. Also, for ${a}_{h}/{a}_{c}\lesssim 0.5$, we obtain ${Q}_{c}\le 0$, ${Q}_{h}\ge 0$ and ${W}_{out}={Q}_{c}+{Q}_{h}\le 0$. In these two regimes of operation of this cycle, we set the work and efficiency to be zero because this work is not inside the area of heat engine.

Comparing Fig. 4 with Fig. 8, show that using the hot coherent thermal bath instead of a thermal bath can improve the total work output and efficiency of the QOHE.

**Figure 9:**
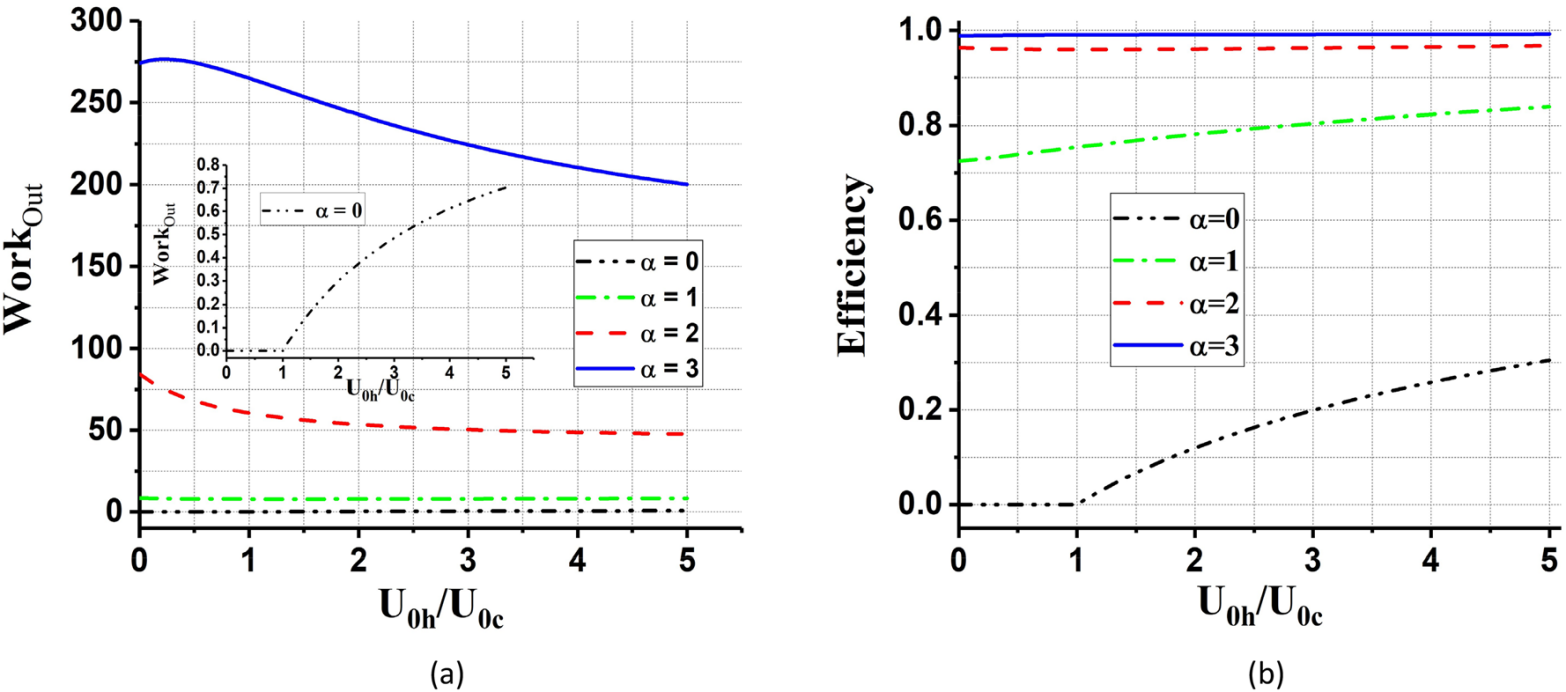
(**a**) The total work output and (**b**) the efficiency (in units of $\hslash \omega ={\hslash }^{2}\lambda {a}^{2}/m$) of the QOHE as a function of potential’s strength, ${U}_{0h}/{U}_{0c}$, for different values of coherent parameter, $\alpha ={0,1},{2,3}$. We set $a={a}_{h}={a}_{c}=1$, and ${\beta }_{h}=0.2{\beta }_{c}$. In the inset of part (**a**) the case $\alpha =0$ is depicted separately.

**Figure 10:**
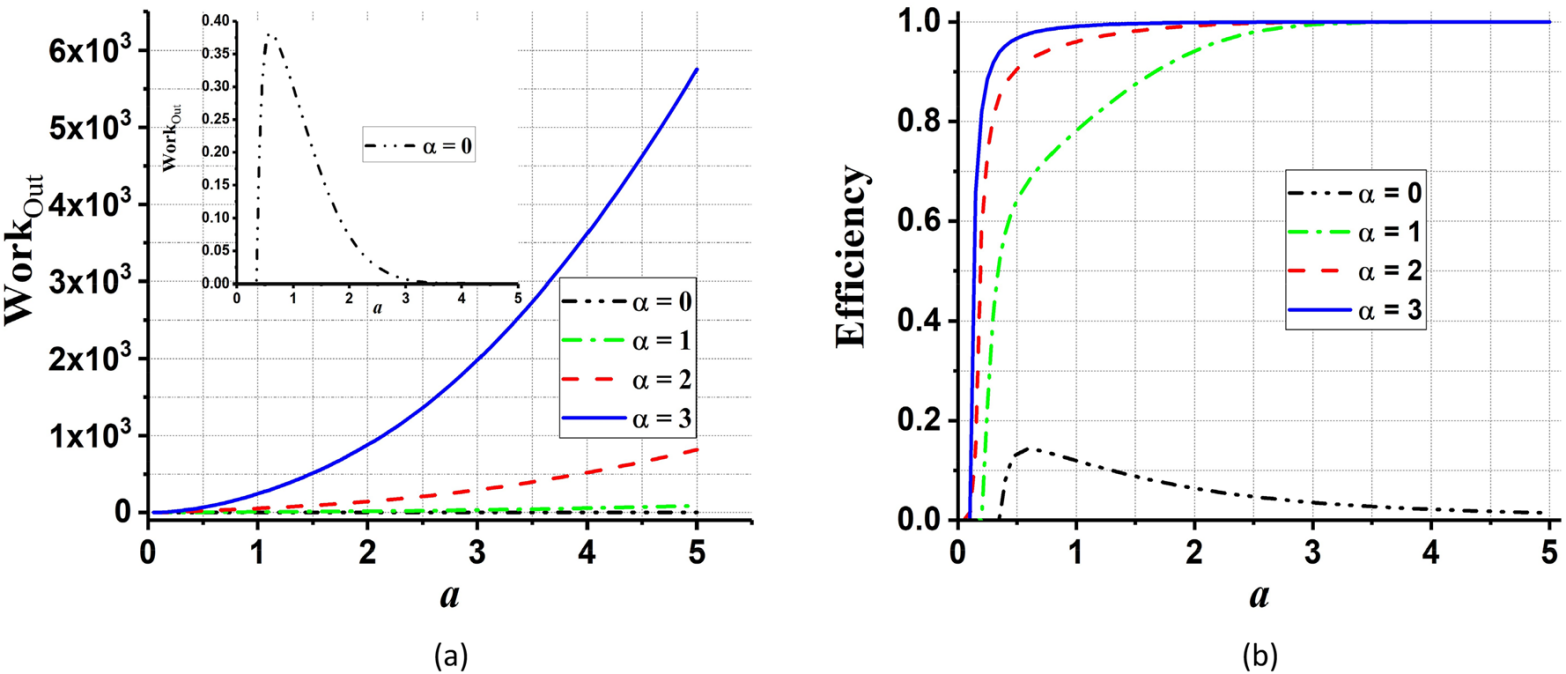
(**a**) The total work output and (**b**) the efficiency (in units of $\hslash \omega ={\hslash }^{2}\lambda {a}^{2}/m$) of the QOHE as a function of $a={a}_{h}={a}_{c}$ for different values of coherent parameter, $\alpha ={0,1},{2,3}$. We set potential’s strength, ${U}_{0h}/{U}_{0c}=2$, and ${\beta }_{h}=0.2{\beta }_{c}$. In the inset of part (**a**) the case $\alpha =0$ is depicted separately.

According to Figs.9 and 10, it can be seen that in the case $\alpha =0$ (the QOHE with normal thermal bath) the total work output and efficiency always are less than $\alpha >0$ (the QOHE with coherent thermal bath). So, increasing the coherence parameter enhances the total work output and efficiency of QOHE.

## Conclusion

In this paper, we investigated the work and efficiency of a particle in the PT potential as a working substance for the COHE and QOHE. We considered two different cases separately. The first case is a QOHE operating between cold and hot thermal baths, and the second case is a QOHE operating between cold thermal and hot coherent thermal baths.

Otto heat engine is a four strokes cycle consisting of two isochoric processes and two adiabatic processes. During the adiabatic processes, the PT potential can be changed by altering parameters ${U}_{0}$, and $a$. The results illustrate that by raising ${a}_{h}/{a}_{c}$ the efficiency of the COHE and QOHE increase.

According to the results, in interval $1.8\le {a}_{h}/{a}_{c}\le 2.4$, the efficiency of the QOHE is more than the efficiency of its classical counterpart. Also, in interval $1.1\le {a}_{h}/{a}_{c}<1.8$, the efficiency of QOHE is not zero, which means low-volume changes can have efficiency, while in this interval, the COHE does not have efficiency. On the other hand, in interval $2.4<{a}_{h}/{a}_{c}\le 2.9$, the efficiency of QOHE is zero, while in this interval, the COHE have efficiency although for ${a}_{h}/{a}_{c}>2.9$ the efficiency of COHE and QOHE are zero.

In the end, we construct the thermal coherent state for a trigonometric PT potential, and then we use this state in the cycle of the QOHE. Our results demonstrate that by using a hot coherent thermal bath, which converts the state of working substance into the thermal coherent state of trigonometric PT potential the work extraction and efficiency of the QOHE improve relative to the classical counterpart, such that the efficiency can reach one.

## Supplementary Information

Supplementary Information.

## Data Availability

The data that support the findings of this study are available within this article.
