# Supplementary material for: Quantum Otto heat engine with Pöschl–Teller potential in contact with coherent thermal bath
Source: Sci Rep. 2023 Jun 29;13:10522. doi: 10.1038/s41598-023-37681-1 (PMC10310849; doi:10.1038/s41598-023-37681-1)
Supplement: Supplementary file 1 — Supplementary Information. [file 41598_2023_37681_MOESM1_ESM.pdf]

Supplementary Information: Quantum Otto heat engine with Pöschl-Teller potential in contact with coherent thermal bath

By: S. Hashemi Abasabadi, S. Y. Mirafzali and H. R. Baghshahi

Appendix A: Calculation of the efficiency of the classical Otto heat engine

In order to calculate the efficiency of the COHE, we use the relation[1],

$$\eta_{Otto} = \frac{W_{out}}{Q_h}. \tag{1}$$

In this relation, $W_{out} = (U_B - U_A) + (U_D - U_C)$ is the total work output where $U_i (i = A, B, C, D)$ is the internal energy of the working substance in point i. The internal energy of the system can be obtained as follows:

$$U = k_B T^2 \frac{\partial}{\partial T} \ln Z, \tag{2}$$

where $Z = \frac{1}{h} \int e^{-\beta H} \mathrm{d}p\mathrm{d}x$ is the partition function. The Hamiltonian of the COHE is $H = \frac{p^2}{2m} + U_0 \tan^2(ax)$. According to this Hamiltonian, the partition function can be calculated as follows:

$$Z = \frac{1}{h} \int_{-\infty}^{+\infty} e^{-\frac{\beta p^2}{2m}} \mathrm{d}p \int_{-\frac{\pi}{2a}}^{+\frac{\pi}{2a}} e^{-\beta U_0 \tan^2(ax)} \mathrm{d}x = \frac{\pi}{ha} \sqrt{\frac{2m\pi}{\beta}} (1 - erf(\sqrt{\beta U_0})) e^{\beta U_0}, \tag{3}$$

where $erf(x)$ is error function. The internal energy of the system is obtained as follows:

$$U = -U_0 + \frac{1}{2\beta} + \frac{\sqrt{\frac{U_0}{\beta\pi}} e^{-\beta U_0}}{1 - erf(\sqrt{\beta U_0})}. \tag{4}$$

From the above relation we can see that in order to calculate the internal energy in points A, B, C and D, temperature of the system in these points must obtained. Temperature in points A and C is definite; $T_A = T_h$ which is the temperature of the hot bath, and $T_C = T_c$ which is the temperature of the cold bath. To specify temperature in the points B and D, we should know the entropy of the system in all points. The entropy of the system can be obtained as follows:

$$S = k_B \ln Z + \frac{U}{T} = \frac{1}{2} k_B \left[1 + \ln\left(\frac{2m\pi^3}{h^2 a^2 \beta} (1 - erf(\sqrt{\beta U_0}))^2 \right) - \frac{2\sqrt{\frac{\beta U_0}{\pi}} e^{-\beta U_0}}{1 - erf(\sqrt{\beta U_0})} \right]. \tag{5}$$

Now using the fact that in the Otto cycle $S_A = S_B$ and $S_C = S_D$, the temperature of the two points B and D can be calculated. As a result, the efficiency of a COHE for a particle in the PT potential can be obtained as follows:

$$\eta_{Otto} = 1 - \frac{(U_B - U_A) + (U_D - U_C)}{U_A - U_D}. \tag{6}$$

Appendix B: Dynamical evolution generated from the coherent thermal bath

Here, we want to demonstrate how the thermal coherent state for the PT potential which is introduced in Eq.(**??**) can be obtained. We consider a particle in the PT potential. The Hamiltonian of this particle is in the following from:

$$H_S = \frac{\hbar\omega}{2} (b^\dagger b + b b^\dagger), \tag{7}$$

where b and $b^\dagger$ are the ladder operator of the PT potential. The dissipation of this particle may be understood from a simple model in which the particle is coupled to a reservoir consists of particles (such as diatomic molecules or quantum dots) subjected to PT potential. The Hamiltonian of the reservoir can be written as follows:

$$H_R = \sum_k \frac{\hbar\Omega_k}{2} (c_k^\dagger c_k + c_k c_k^\dagger), \tag{8}$$

where c and $c_k^\dagger$ are the ladder operator of the k'th particle in the reservoir. Also, the interaction Hamiltonian between the particle and the reservoir can be expressed as:

$$H_{S-R} = \sum_k i g_k (b c_k^\dagger - b^\dagger c_k), \tag{9}$$

where g_k is the coupling constant between particle and k'th particle in the reservoir. Now, let us suppose that the reservoir is initially considered as:

$$\rho_R = \prod_k Z_R^{-1} D_k(\zeta) e^{(-\beta\hbar\Omega_k(c_k^\dagger c_k + c_k c_k^\dagger)/2)} D_k^\dagger(\zeta). \tag{10}$$

Under the Born-Markov approximation the dynamics of the particle can be described using the following Lindblad master equation in the interaction picture:

$$\dot{\rho}_s(t) = \mathscr{L}(\rho_s(t)) = \sum_{i=\pm} R_i \rho_s(t) R_i^\dagger - \frac{1}{2}\{R_i^\dagger R_i, \rho_s(t)\}, \tag{11}$$

where $R_- = \sqrt{\gamma\langle (n+1) f^2(n+1)\rangle} R$ and $R_+ = \sqrt{\gamma\langle n f^2(n)\rangle} R^\dagger$, with $R = D(\zeta) a D^\dagger(\zeta)$. Also $D(\zeta)$ is the displacement operator for the PT potential (Eq.(**??**)), γ is the spontaneous emission rate, $f^2(n) = (2\lambda + n - 1)/2\lambda$ and $\langle n f^2(n)\rangle = \sum_n n f^2(n) \exp(-\beta E_{n,\lambda}) Z^{-1}$. The steady state solution of the above Lindblad equation is thermal coherent state for PT potential as follows:

$$\rho(\zeta,\lambda,\beta) = D(\zeta)\frac{e^{-\beta H_s}}{Z} D^\dagger(\zeta), \tag{12}$$

where $Z = Tr\left[e^{-\beta H_s}\right]$ is partition function i.e. $\mathscr{L}(\rho(\zeta,\lambda,\beta)) = 0$. So, at large enough time the state of the particle becomes the desired thermal coherent state.

Appendix C: The average energy at point A for the quantum Otto cycle with coherent thermal bath

Here, we demonstrate the calculation of the average energy at point A, Eq.(10) of the manuscript in detail. The Hamiltonian is $H_h = \frac{\hbar\omega}{2}(b^\dagger b + b b^\dagger)$. The average energy at point A is $\langle H_h\rangle_A = \mathrm{Tr}(\rho_A H_h)$, where $\rho_A = D(\alpha) Z_h^{-1} \exp(-\beta_h H_h) D^\dagger(\alpha)$ is the density matrix at point A and $D^\dagger(\alpha) = \exp(\alpha b^\dagger - \alpha^* b)$ is the displacement operator for the Pöschl-Teller potential. According to the expression of ρ_A in Eq.(7) and using Eq.(5) of the manuscript, the first expression in Eq.(10) of the manuscript for $\langle H_h\rangle_A$ can be obtained. The second expression of $\langle H_h\rangle_A$ in Eq.(10) of the manuscript can be obtained as follows:
We can write,

$$\langle H_h\rangle_A = \mathrm{Tr}\big(D(\alpha) Z_h^{-1} \exp(-\beta_h H_h) D^\dagger(\alpha) H_h\big) = \mathrm{Tr}\big(Z_h^{-1} \exp(-\beta_h H_h) D^\dagger(\alpha) H_h D(\alpha)\big). \tag{13}$$

Now, we calculate $D^\dagger(\alpha) H_h D(\alpha) = \frac{\hbar\omega}{2} D^\dagger(\alpha)(b^\dagger b + b b^\dagger) D(\alpha)$. First we should obtain $D^\dagger(\alpha) b D(\alpha)$ and $D^\dagger(\alpha) b^\dagger D(\alpha)$. By using the Baker-Hausdorff formula, $e^X Y e^{-X} = Y + [X,Y] + \frac{1}{2!}[X,[X,Y]] + \ldots$, and also by applying the commutation relations between $b, b^\dagger$, and b_0 ($[b, b^\dagger] = b_0$, $\quad [b, b_0] = \frac{1}{\lambda} b$, $\quad [b^\dagger, b_0] = -\frac{1}{\lambda} b^\dagger$)[2] we can obtain,

$$\begin{aligned} D^\dagger(\alpha) b D(\alpha) =& e^{\alpha b^\dagger - \alpha^* b} b e^{\alpha^* b - \alpha b^\dagger} \\ =& b + \alpha b_0 + \frac{1}{2!}\Big(\frac{|\alpha|^2}{\lambda} b + \frac{\alpha^2}{\lambda} b^\dagger\Big) + \frac{2}{3!}\frac{\alpha|\alpha|^2}{\lambda} b_0 + \frac{2}{4!}\Big(\frac{|\alpha|^4}{\lambda^2} b + \frac{\alpha^2|\alpha|^2}{\lambda^2} b^\dagger\Big) \\ &+ \frac{4}{5!}\frac{\alpha|\alpha|^4}{\lambda^2} b_0 + \frac{4}{6!}\Big(\frac{|\alpha|^6}{\lambda^3} b + \frac{\alpha^2|\alpha|^4}{\lambda^3} b^\dagger\Big) + \frac{8}{7!}\frac{\alpha|\alpha|^6}{\lambda^3} b_0 + \ldots, \\ =& \Big(1 + \frac{1}{2!}\frac{|\alpha|^2}{\lambda} + \frac{2}{4!}\frac{|\alpha|^4}{\lambda^2} + \frac{4}{6!}\frac{|\alpha|^6}{\lambda^3} + \ldots\Big) b + \Big(\frac{1}{2!}\frac{\alpha^2}{\lambda} + \frac{2}{4!}\frac{\alpha^2|\alpha|^2}{\lambda^2} + \frac{4}{6!}\frac{\alpha^2|\alpha|^4}{\lambda^3} + \ldots\Big) b^\dagger \\ &+ \Big(\alpha + \frac{2}{3!}\frac{\alpha|\alpha|^2}{\lambda} + \frac{4}{5!}\frac{\alpha|\alpha|^4}{\lambda^2} + \frac{8}{7!}\frac{\alpha|\alpha|^6}{\lambda^3} + \ldots\Big) b_0, \\ =& \frac{1}{2}\Big(\cosh\big(\sqrt{\frac{2}{\lambda}}|\alpha|\big) + 1\Big) b + \frac{\alpha^2}{2|\alpha|^2}\Big(\cosh\big(\sqrt{\frac{2}{\lambda}}|\alpha|\big) - 1\Big) b^\dagger + \frac{\alpha}{|\alpha|}\sqrt{\frac{\lambda}{2}} \sinh\big(\sqrt{\frac{2}{\lambda}}|\alpha|\big) b_0. \end{aligned} \tag{14}$$

And also we have,

$$D^{\dagger}(\alpha)b^{\dagger}D(\alpha) = \Big(D^{\dagger}(\alpha)bD(\alpha)\Big)^{\dagger} = \frac{1}{2}\big(\cosh(\sqrt{\frac{2}{\lambda}}|\alpha|)+1\big)b^{\dagger} + \frac{\alpha^{*2}}{2|\alpha|^2}\big(\cosh(\sqrt{\frac{2}{\lambda}}|\alpha|)-1\big)b + \frac{\alpha^{*}}{|\alpha|}\sqrt{\frac{\lambda}{2}}\sinh(\sqrt{\frac{2}{\lambda}}|\alpha|)b_0. \tag{15}$$

So we obtain,

$$\langle H_h\rangle_A = \Big(\frac{1}{2}\cosh^2(\sqrt{\frac{2}{\lambda}}|\alpha|)+\frac{1}{2}+\sinh^2(\sqrt{\frac{2}{\lambda}}|\alpha|)\Big)\mathrm{Tr}\Big(Z_h^{-1}\exp(-\beta_h H_h)H_h\Big) + \frac{\hbar^2 a^2}{2m}(\lambda^2-\lambda)\sinh^2(\sqrt{\frac{2}{\lambda}}|\alpha|). \tag{16}$$

References

1. Sayyaadi, H. *Modeling, Assessment, and Optimization of Energy Systems* (Academic Press, 2020).
2. Román-Ancheyta, R., De los Santos-Sanchez, O. & Récamier, J. Ladder operators and coherent states for nonlinear potentials. *J. Phys. A* **44**, 435304 (2011).
